# Nutritional assessments in pregnancy and the risk of postpartum depression in Chinese women: A case-control study

**DOI:** 10.1097/MD.0000000000021647

**Published:** 2020-08-14

**Authors:** Dan Shi, Guo-hua Wang, Wen Feng

**Affiliations:** Department of Obstetrics and Gynecology, the First People's Hospital of Lianyungang, Jiangsu, P.R. China.

**Keywords:** case-control, food frequency questionnaire, nutritional assessment, postpartum depression

## Abstract

Prevalence of postpartum depression (PD) in Chinese women is rising and its associated factors are not well known. In this study we aim to explore the associations between nutritional factors in pregnancy and the risk of PD in Chinese women.

A case-control study was performed in our hospital during January 2016 to June 2019. A food frequency questionnaire was designed to collect food consumption before the childbirth preceding month. Nutrition related biochemical indicators including fasting blood-glucose (GLU), total cholesterol (TC), triglyceride (TG), low density lipoprotein (LDL), high density lipoprotein (HDL), and uric acid in the third trimester of pregnancy were detected. Logistic regression model was applied to compute odds ratio (OR) and its corresponding 95% confidence interval (CI).

There were 565 participants in this study, which comprised 182 individuals with PD and 383 individuals without. Patients with PD had higher odds of increased GLU (OR=2.62, 95%CI = 1.67–4.11), TC (OR = 1.73 95%CI = 1.22–2.46), TG (OR = 2.43, 95%CI = 1.55–3.81), and LDL (OR = 3.41, 95%CI = 2.09–5.57), but decreased HDL (OR = 3.41, 95%CI = 2.09–5.57) during pregnancy. With respect to uric acid, there was lack of no statistical association (OR = 2.23, 95%CI = 0.82–6.26). Food frequency questionnaire indicated a higher meat intake, but a lower vegetable, fruit, fish, and poultry intake in patients with PD during pregnancy.

Increased GLU, TC, TG, and LDL, but decreased HDL in later stages of pregnancy might be associated with PD.

## 1 Introduction

Postpartum depression (PD) is one of the commonest psychological problems to affect postpartum women's health. Its prevalence rate worldwide was reported to be 11.9% and a higher rate of developing countries.^[1,2]^ PD could develop symptoms as depressed emotion, fatigue, and even suicidal behavior.^[1,3]^ Mothers with PD were associated with autistic disorder and developmental retardation in their offspring.^[3,4]^ Thus, it is urgent to prevent pregnant women from PD.

Nutrition during pregnancy is a vital factor of PD. Accumulating evidence has proved that deficiencies in unsaturated fatty acids and vitamin D were more commonly in patients with PD.^[5,6]^ The possible mechanism might be involved in regulation of proinflammatory cytokines and serotonergic neurotransmitters.^[6]^ Physiological increases in nutrition related biochemical indicators, such as blood glucose, lipid profile, and uric acid (UA), are reported during pregnancy. Some previous studies have reported an association between serum lipid concentrations and the risk of depression,^[5,7]^ while its mechanisms were not well understood. Some other studies demonstrated an increased risk of anxiety and depression in postpartum women who had a rapid reduction in the concentrations of these biochemical indicators.^[8]^ The physiological increases may vary in pregnant women from different regions.

Chinese economy has enjoyed a rapid development over the past 3 decades and the lifestyle in Chinese people is changing dramatically, especially reflected on food consumption. An increased intake of animal-based foods, but a decreased intake of plant-based foods is characterized as modern Chinese.^[9–11]^ In this study, we conducted a case-control study to explore the association between nutrition assessments during pregnancy and the risk of PD in Chinese women.

## 2 Method

### 2.1 Study design

This study was hospital-based case-control designed with a study period from January 2016 to June 2019. Participants with PD were recruited in the case group according to the criteria as follows:

(1) first time diagnosis for PD;
(2) singleton and term delivery;
(3) without other postpartum disease;
(4) without severe co-morbidities including neoplasm, cardiovascular disease, mental disorder, or severe infection during pregnancy;
(5) no smoking or drinking during pregnancy;
(6) age from 20 to 40 years.
  The controls were selected from puerpera who had childbirth in our hospital as the same period of the cases and were free of PD. Protocol of this study was in accordance with the Declaration of Helsinki and the research ethics principles of the Committee of our hospital. Eligible participants attended this study with signed informed consent.

Baseline information was extracted from medical records of our hospital information system, including maternal age, maternal weight, neonatal weight, gestational weeks, neonatal gender, type of delivery.

### 2.2 PD

The Chinese version of Edinburgh postnatal depression scale (EPDS) was employed to identify PD,^[12]^ which has a fine reliability and validity and has been widely applied in China.^[13–15]^ EPDS contained 10 items including mood, pleasure, remorse, anxiety, fear, coping ability, insomnia, sadness, crying, and NSSI.^[12]^ Score in each item range of 0 to 3 points.^[12]^ Puerpera obtained an EPDS score ≥10 points to suggest the presence of PD, while an EPDS score < 10 points to suggest free of PD.^[12]^

### 2.3 Food consumption during pregnancy

A food frequency questionnaire (FFQ) was designed to collect food consumption before the childbirth preceding month. Stable food (rice, wheat, other), vegetable (light, dark), fruit, fish, meat (pork, beef, and mutton, other red meat), poultry, and water were list on the FFQ (8, 9, 31). Participants were interviewed by well-trained investigators. Frequency and amount to food consumption before the childbirth preceding month were recorded. Several options were applied for food frequency, including never, times per day, times per week, and times per month.

### 2.4 Biochemical indicators detection during pregnancy

A routine fasting blood sample (5–10 mL) was collected from each participant in the third trimester of pregnancy. The samples were stored at −80°C until used. Biochemical indicators including fasting blood-glucose (GLU), total cholesterol (TC), triglyceride (TG), low density lipoprotein (LDL), HDL, and UA were detected by valid methods as described previously.^[5,7]^ Dyslipidemia in pregnant women was diagnosed when TC, TG, LDL concentrations were above the 95th percentile, and HDL concentration was below the 5th percentile for gestational age.^[16]^ Hyperglycemia in pregnant women was defined as GLU ≥7.0 mmol/L.^[17]^ Hyperuricemia was defined as UA ≥ 390 μmol/L.^[18,19]^

### 2.5 Statistical analysis

R version 3.5.1 (The R Foundation, Vienna, Austria) was applied for statistical analysis. Categorical variables were given as frequency and percentile, while continuous variables were given as mean and standard deviation. Based on variable types, Student *t* test or Chi-squared test was used to evaluate variable difference in the 2 groups. A logistic regression model was applied to evaluate the association between nutrition related biochemical indicators (GLU, TC, TG, LDL, HDL, and UA) and PD. Crude odds ratio (OR) and its corresponding 95% confidence interval (CI) were computed. After then, adjusted ORs were calculated by adjusting for several possible risk factors (maternal age, maternal weight, neonatal weight, gestational weeks, neonatal gender, delivery type, systolic blood pressure, and diastolic blood pressure). A *P*-value < .05 was set to describe statistical difference.

## 3 Results

### 3.1 Characteristics of eligible participants

**Figure 1:**
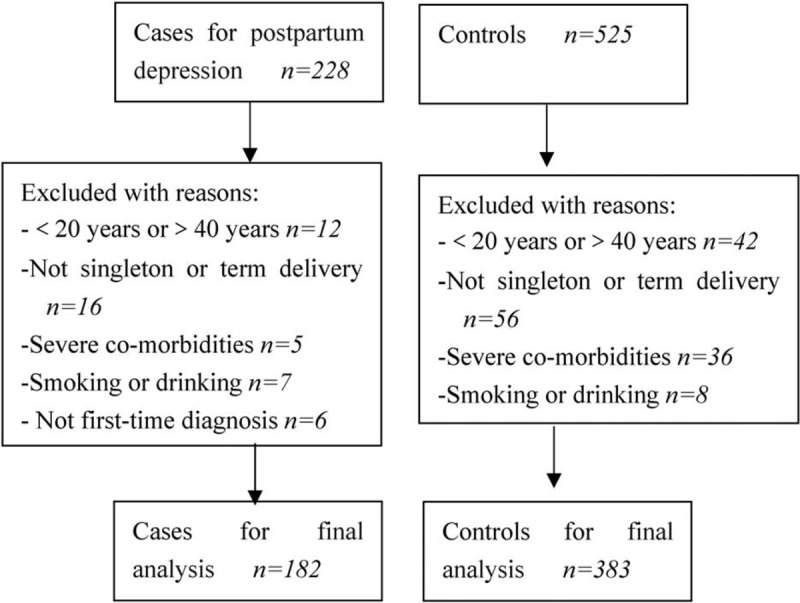
Flowchart exhibiting the selection of eligible individuals.

A total of 565 participants were eligible in this study, which comprised 182 puerpera with PD and 383 puerpera without. Flowchart exhibiting participant selection is showed in Figure 1. Average age in case group was 31.9 ± 5.3 and 31.5 ± 5.7 years in control group. Vaginal delivery and caesarean section delivery were 68.1% and 31.9% in case group, and 63.4% and 36.6% in control group. Other baseline information about eligible participants is summarized in Table 1.

**Table 1:**
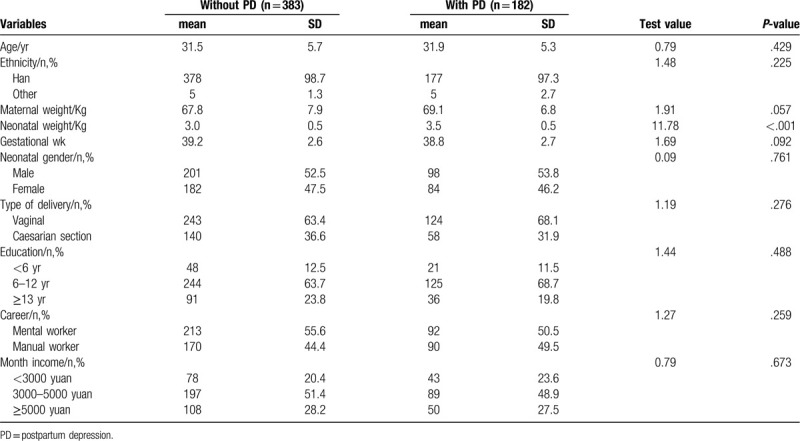
Characteristics of eligible participants with postpartum depression and without.

### 3.2 Association between pregnant biochemical indicators and PD

GLU in case group (5.0 ± 1.0 mmol/L) was significantly higher than the control group (5.3 ± 2.1 mmol/L). Logistics analysis demonstrated that patients with PD have a 2.67-fold (95%CI = 1.67–4.11, *P*-value = .012) higher odds exposed to elevated GLU level. After adjusting for several possible risk factors, the result remained.

Serum lipid indicators including TC, TG as well as LDL in case group were also statistically higher than the control group, but HDL was found to be lower in case group. Compare with the controls, ORs for increased TC, TG, and LDL during pregnancy were 1.73 (95%CI = 1.22–2.46, *P*-value = .023), 2.43 (95%CI = 1.55–3.81, *P*-value <.001), and 3.41 (95%CI = 2.09–5.57, *P*-value < .001), respectively in patients with PD. Additionally, an inverse association between decreased HDL and PD (OR = 3.41, 95%CI = 2.09–5.57, *P*-value < .001) was identified. The positive associations mentioned above remained after matching with some risk factors.

**Figure 2:**
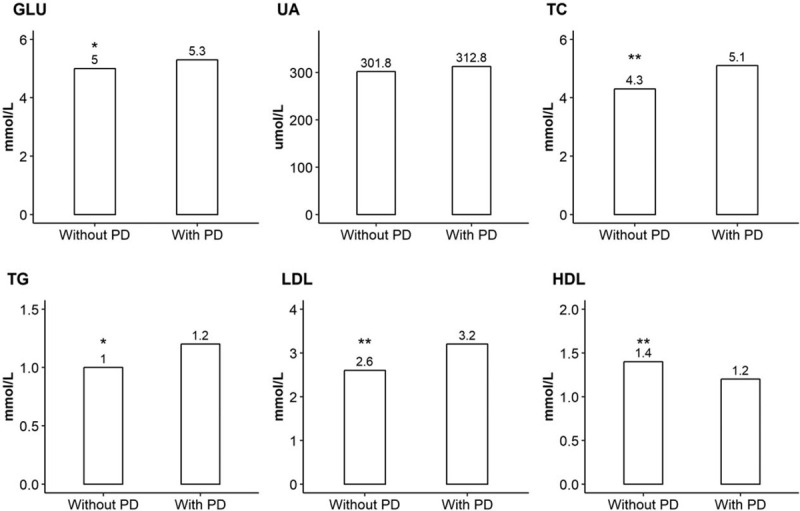
Comparison of nutrition biomarkers between participants with postpartum depression and without postpartum depression. ^∗^=*P*-value < .05, ^∗∗^=*P*-value < .01, GLU = GLU, HDL = high density lipoprotein, LDL = low density lipoprotein, TC = total cholesterol, TG = triglyceride, UA = uric acid.

However, with respect to UA, no statistically association was identified (OR = 2.23, 95%CI = 0.82–6.26, *P*-value = .137). Detail information is shown in Figure 2 and Table 2.

**Table 2:**
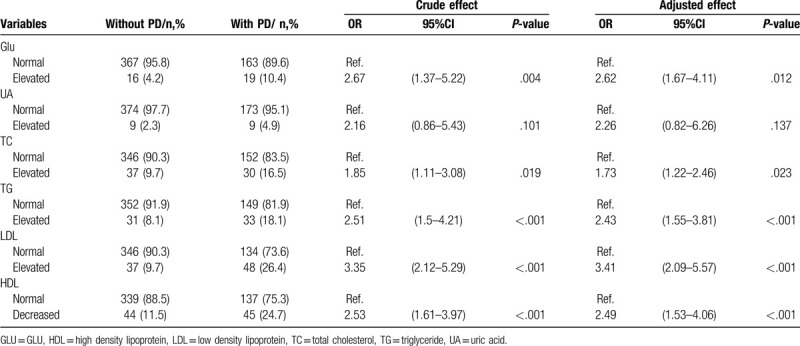
Association between pregnant related biochemical indicators and postpartum depression.

### 3.3 Food consumption before the childbirth preceding month

According to FFQ, patients with PD were found to have a higher meat intake (142.9 ± 57.7 g/d) before the childbirth preceding month when compared to those free of PD (121.0 ± 65.8 g/d), while lower consumptions of vegetable, fruit, fish, and poultry were characterized in patients with PD. There were lack material difference about stable food, water, and energy intake between the 2 group. Detail information is demonstrated in Table 3.

**Table 3:**
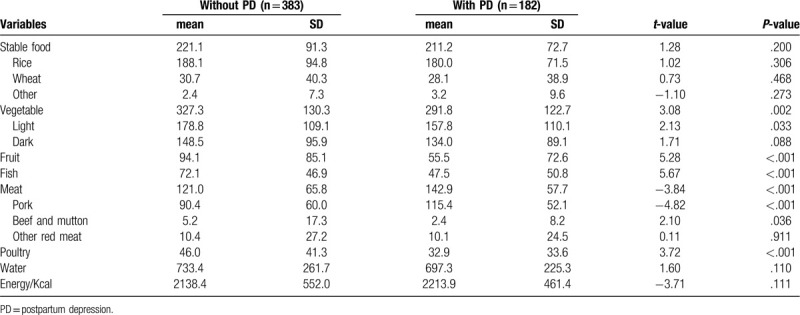
Various kinds of food consumption (g/d) between participants with postpartum depression and without postpartum depression.

## 4 Discussion

In this hospital-based case-control study, women with postnatal depression were found to have higher odds of elevated GLU, TC, TG, and LDL, but decreased HDL in later stages of pregnancy when compared with parturient free of PD. These findings were robust as the associations remained significant after adjusting for possible confounders. What is more, diet pattern of women with PD was different from those without. Women with PD were characterized as higher meat consumption, but lower vegetable, fruit, fish, and poultry consumption during later pregnancy.

Gestational diabetes mellitus would receive much attention due to its adverse effects on maternal and offspring. In this study, a higher OR of increased GLU was found in women with postnatal depressive symptoms. Mak et al^[20]^ also proved the positive association between gestational diabetes and the risk of postnatal depression through a prospective cohort study comprising 1449 mothers. Our results were in accordance with possibly biological plausibility that abnormal regulation of blood glucose regulation during pregnancy may increase susceptibility to occurrence of PD through activating and releasing cortisol and inflammatory adipokine. Function of hypothalamic-pituitaryadrenal axis in patients with PD were reported to be irregular and an increased level of cortisol was observed, which could partly attribute to abnormal blood glucose or diabetes.^[21,22]^ Irregular blood glucose was also proved to be involved in activation and secretion of inflammation and adipokine, such as Interleukin-6, which are known to be associated with elevated risk of PD.^[23,24]^ In addition, a previous study indicated that symptoms and adverse birth outcomes associated with abnormal blood glucose might bring additional psychological burden and stress to these mothers.^[20]^

The current study also supported the material associations between dyslipidemia during pregnancy and the risk of postnatal depression. Among several potential mechanisms documented in previous publications, the role of serum lipids on serotonin attracts the most attention. Serotonin is a neurotransmitter and plays a vital role in adjusting controlling appetite, sleep, and mood.^[25]^ The fluctuations in serum lipids, such as reduction of cholesterol, would affect normal structure of brain cell including reduction of cholesterol in cell membranes and lipid micro viscosity (23). Without to integrate structure, function of serotonin receptors on brain cell would be damaged, such as impairing its uptake function of serotonin.^[26]^ Reduced serotonin in brain was proved to be associated with depression.^[27]^

Another possible mechanism regarding the association between serum lipids and PD may be explained by hypothalamic-pituitaryadrenal axis. Neurochemical functions, such as synthesis and used of norepinephrine, dopamine, and serotonin in patients with depressive disorders are abnormal, which may attribute to dysregulation adrenocorticotropin hormone and elevating cortisol and cortisone levels.^[28,29]^ Previous study confirmed a material association between cortisol excretion rate and serum lipids, indicative of an indirect effect of serum lipids on development of depression.^[8,30]^ More effectors are needed to explore the biological mechanisms about this association.

Our findings suggest a higher meat consumption, but lower vegetable, fruit, fish, and poultry consumption in women with PD before the childbirth preceding month. To supported growth of fetal and infant, women during pregnancy, or lactation should intake much more nutrients, in particular the essential nutrients, such as n-3 fatty acids and high-quality protein.^[7]^ An increasing evidence had confirmed a significant association between food consumption and development of depression.^[31,32]^ A higher meat intake may be associated with low-grade inflammation, such as elevated C-protein, which have an involvement in the pathogenesis of depression.^[33]^ Vegetable and fruit contain abundant antioxidants, such as vitamin C and anthocyanin, which exert beneficial protective effects on against depressive disease.^[34,35]^ Besides, vegetable and fruit could also provide dietary fiber and block gut microbiota into blood circulation which may have adverse effect on central nervous system.^[36–38]^ The protective effect of poultry and fish intake against depression may be owing to their high content of polyunsaturated fatty acids.^[34]^ These fatty acids have important anti-inflammatory properties and exert irreplaceable role in brain function and serotonin neurotransmission, such as the fluidity of neurons cell membrane.^[31]^ Additionally, fatty acids especially n-3 fatty acids, were thought to improve mood and cognition in patients with major depression.^[34]^

Findings from this study should be considered in view of some limitations. To start with, this study was case-control design, which could provide clue of risk factors for PD, but cannot draw a causal association between nutritional assessments in pregnancy and the risk of PD due to its reverse-chronological order inference of disease and cause. Secondly, bias from observational study cannot be eliminated, in particular recall bias. Although we performed a multi-factor logistic regression model to adjust the effects of several potential risk factors, some other possible confounding factors might exist. Thirdly, the sample size is small and participants were from a local place in China, indicating the limitation of generalizability.

## 5 Conclusion

In this study, women with postnatal depression were found to have higher odds of elevated GLU, TC, TG, and LDL, but decreased HDL in later stages of pregnancy. Diet pattern of women with PD were different from those without. Women with PD were characterized with higher meat consumption, but lower vegetable, fruit, fish, and poultry consumption during later pregnancy. As observational study cannot draw a causal link, further well-designed perspective and experimental studies are warranted.

## Author contributions

**Conceptualization:** Wen Feng.

**Data curation:** Dan Shi, Wen Feng.

**Formal analysis:** Guo-hua Wang.

**Funding acquisition:** Wen Feng.

**Investigation:** Dan Shi.

**Methodology:** Guo-hua Wang, Wen Feng.

**Resources:** Wen Feng.

**Software:** Guo-hua Wang.

**Supervision:** Wen Feng.

**Writing – original draft:** Dan Shi, Wen Feng.

**Writing – review & editing:** Guo-hua Wang.
